# Supplementary material for: Determinants for α4β2 vs. α3β4 Subtype Selectivity of Pyrrolidine-Based nAChRs Ligands: A Computational Perspective with Focus on Recent cryo-EM Receptor Structures
Source: Molecules. 2021 Jun 12;26(12):3603. doi: 10.3390/molecules26123603 (PMC8231201; doi:10.3390/molecules26123603)
Supplement: Supplementary file 1 [file molecules-26-03603-s001.zip › molecules-1228023-supplementary.pdf]

Supplemental Material

Determinants for α4β2 vs. α3β4 Subtype Selectivity of Pyrrolidine-Based nAChRs Ligands: A Computational Perspective with Focus on Recent cryo-EM Receptor Structures

Francesco Bavo [1,2], Marco Pallavicini [1], Rebecca Appiani [1] and Cristiano Bolchi [1,*]

[1] Dipartimento di Scienze Farmaceutiche, Universita'degli Studi di Milano, I-20133 Milano, Italy; francesco.bavo@sund.ku.dk (F.B.); marco.pallavicini@unimi.it (M.P.); rebecca.appiani@unimi.it (R.A.)
[2] Department of Drug Design and Pharmacology, University of Copenhagen, DK-2100 Copenhagen, Denmark
* Correspondence: cristiano.bolchi@unimi.it

Table of contents

**Table S1**	Overview of the relevant interactions of the compounds (S)-**nicotine**, **A-84543** and **AT-1001** reported in Figure 4.
**Table S2**	Overview of the relevant interactions of the compounds (S)-**3-7** reported in Figure 5 and 6.
**Table S3**	Overview of the relevant interactions of the compounds (S)-**8-14** reported in Figure 7.
**Table S4**	Overview of the relevant interactions of the compounds (*S*)-**15** and (*S*)-**16** reported in Figure 8.
**Table S5**	Overview of the relevant interactions of the compounds (*S*,*R*)-**2**, (*S*,*R*)-**26,** (*S*,*R*)-**29,** (*S*,*R*)-**31** and (*S*,*S*)-**37-40** reported in Figure 9 and Figure 11, respectively.
**Table S6**	Overview of the relevant interactions of the compounds (*S*,*R*)-**33-36** reported in Figure 10.
**Table S7**	RMSD values from the self-docking study.

Table S1. Overview of the relevant interactions of the compounds (S)-**nicotine**, **A-84543** and **AT-1001** reported in Figure 4. The substructure of the residue involved in the interaction is reported in brackets. The atom of the ligand interacting with each residue is highlighted in bold. The type of interaction is reported in brackets. Interactions marked with * are not explicitly visualized, but at a suitable distance for interaction, very close to the default visualization thresholds (< 7 Å for π/+ and < 3.2 Å for H-bonds).

6cnj-wc-hα4β2	**hα4**				**Water**	**hβ2**			
Compound	**Tyr197 (ring)**	**Tyr204 (ring/OH)**	**Trp156 (ring)**	**Trp156 (CO)**		**Trp57 (ring)**	**Asn109 (CO)**	**Phe119 (ring)**	**Leu121 (NH)**
(*S*)-**nicotine**	N^+ (π/+)*	N^+ (π/+)	N^+ (π/+)	**NH** (HB)	$\mathbf{N}_{pyr}$ (HB)	N^+ (π/+)	$\mathbf{H}_2$O (HB)	Pyr (π/π)*	H_2**O** (HB)
**A-84543**	N^+ (π/+)*	N^+ (π/+)	N^+ (π/+)	**NH** (HB)*	$\mathbf{N}_{pyr}$ (HB)	N^+ (π/+)*	$\mathbf{H}_2$O (HB)	Pyr (π/π)	H_2**O** (HB)

6pv7-wc-hα3β4	**hα3**				**Water**	**hβ4**			
Compound	**Tyr190 (ring)**	**Tyr197 (ring)**	**Trp149 (ring)**	**Trp149 (CO)**		**Trp59 (ring)**	**Asn111 (CO)**	**Leu121 (s.chain)**	**Leu123 (NH)**
(*S*)-**nicotine**	N^+ (π/+)*	N^+ (π/+)	N^+ (π/+)	**NH** (HB)	$\mathbf{N}_{pyr}$ (HB)	N^+ (π/+)	$\mathbf{H}_2$O (HB)	-	H_2**O** (HB)
**A-84543**	N^+ (π/+)*	N^+ (π/+)	N^+ (π/+)	**NH** (HB)*	$\mathbf{N}_{pyr}$ (HB)*	N^+ (π/+)	$\mathbf{H}_2$O (HB)	Pyr (VdW)	H_2**O** (HB)

6pv7-wc-rα3β4	**hα3**					**hβ4**			
Compound	**Tyr190 (ring)**	**Tyr197 (ring)**	**Trp149 (ring)**	**Trp149 (CO)**	**Water**	**Trp59 (ring)**	**Asn111 (CO)**	**Asn121 (s.chain)**	**Leu123 (NH)**
(*S*)-**nicotine**	N^+ (π/+)*	N^+ (π/+)	N^+ (π/+)	**NH** (HB)	$\mathbf{N}_{pyr}$ (HB)	N^+ (π/+)	$\mathbf{H}_2$O (HB)	-	H_2**O** (HB)
**A-84543**	N^+ (π/+)*	N^+ (π/+)	N^+ (π/+)	**NH** (HB)	-	N^+ (π/+)	$\mathbf{H}_2$O (HB)	-	H_2**O** (HB)

6pv8-rα3β4	**hα3**				**hβ4**			
Compound	**Tyr190 (ring)**	**Tyr197 (ring)**	**Trp149 (ring)**	**Trp149 (CO)**	**Trp59 (ring)**	**Asn111 (CO)**	**Asn121 (s.chain)**	**Leu123 (NH)**
**AT-1001** (AB)	N^+ (π/+)	N^+ (π/+)	N^+ (π/+)	**NH** (HB)	N^+ (π/+)	-	Br (clash)	-
**AT-1001** (DE)	N^+ (π/+)*	N^+ (π/+)	N^+ (π/+)	**NH** (HB)	N^+ (π/+)	-	-	-

Table S2. Overview of the relevant interactions of the compounds (S)-**3-7** reported in Figure 5 and 6. The substructure of the residue involved in the interaction is reported in brackets. The atom of the ligand interacting with each residue is highlighted in bold. The type of interaction is reported in brackets. Interactions marked with * are not explicitly visualized, but at a suitable distance for interaction, very close to the default visualization thresholds (< 7 Å for π/+ and < 3.2 Å for H-bonds).

6cnj-wf-hα4β2	**hα4**				**hβ2**			
Compound	**Tyr197 (ring)**	**Tyr204 (ring)**	**Trp156 (ring)**	**Trp156 (CO)**	**Trp57 (ring)**	**Asn109 (CO)**	**Phe119 (ring)**	**Leu121 (NH)**
(*S*)-**3**	N$^+$ (π/+)*	N$^+$ (π/+)	N$^+$ (π/+)	N**H** (HB)	N$^+$ (π/+)	O**H** (HB)	Phe (π/π)	**O**H (HB)
(*S*)-**4**	N$^+$ (π/+)*	N$^+$ (π/+)	N$^+$ (π/+)	N**H** (HB)	N$^+$ (π/+)*	-	Phe (π/π)*	-
(*S*)-**5**	N$^+$ (π/+)*	N$^+$ (π/+)	N$^+$ (π/+)	N**H** (HB)*	N$^+$ (π/+)*	-	Phe (π/π)*	O**Me** (clash)
(*S*)-**6**	N$^+$ (π/+)*	N$^+$ (π/+)	N$^+$ (π/+)	N**H** (HB)	N$^+$ (π/+)	-	Phe (π/π)*	N**O**$_2$ (HB)
(*S*)-**7**	N$^+$ (π/+)*	N$^+$ (π/+)	N$^+$ (π/+)	N**H** (HB)	N$^+$ (π/+)*	O**H** (HB)	Phe (π/π)	**O**H (HB)

6pv7-wf-hα3β4	**hα3**				**hβ4**			
Compound	**Tyr190 (ring)**	**Tyr197 (ring)**	**Trp149 (ring)**	**Trp149 (CO)**	**Trp59 (ring)**	**Asn111 (CO)**	**Leu119 (s.chain)**	**Leu123 (NH)**
(*S*)-**3**	N$^+$ (π/+)*	N$^+$ (π/+)	N$^+$ (π/+)	N**H** (HB)	N$^+$ (π/+)	O**H** (HB)	Phe (VdW)	**O**H (HB)*
(*S*)-**7**	N$^+$ (π/+)*	N$^+$ (π/+)	N$^+$ (π/+)	N**H** (HB)	N$^+$ (π/+)	O**H** (HB)	Pyr (VdW)	**O**H (HB)*

6cnj-wc-hα4β2	**hα4**				**Water**	**hβ2**			
Compound	**Tyr197 (ring)**	**Tyr204 (ring/OH)**	**Trp156 (ring)**	**Trp156 (CO)**		**Trp57 (ring)**	**Asn109 (CO)**	**Phe119 (ring)**	**Leu121 (NH)**
(*S*)-**7**	N$^+$ (π/+)*	N$^+$ (π/+)<br>**OH**$_{pyr}$ (HB)	N$^+$ (π/+)	N**H** (HB)	**N**$_{pyr}$ (HB)	N$^+$ (π/+)	**H**$_2$O (HB)	Pyr (π/π)	H$_2$**O** (HB)

6pv7-wc-hα3β4	**hα3**				**Water**	**hβ4**			
Compound	**Tyr190 (ring)**	**Tyr197 (ring)**	**Trp149 (ring)**	**Trp149 (CO)**		**Trp59 (ring)**	**Asn111 (CO)**	**Leu121 (s.chain)**	**Leu123 (NH)**
(*S*)-**7**¤	N$^+$ (π/+)*	N$^+$ (π/+)	N$^+$ (π/+)	N**H** (HB)	-	-	**H**$_2$O (HB)	-	H$_2$**O** (HB)

¤ The hydroxyl group of (S)-7 establish an additional H-bond with the side chain of hα3-Ser150

Table S3. Overview of the relevant interactions of the compounds (S)-**8-14** reported in Figure 7. The substructure of the residue involved in the interaction is reported in brackets. The atom of the ligand interacting with each residue is highlighted in bold. The type of interaction is reported in brackets. Interactions marked with * are not explicitly visualized, but at a suitable distance for interaction, very close to the default visualization thresholds (< 7 Å for π/+ and < 3.2 Å for H-bonds).

2ifd-wf-hα4β2	**hα4**				**hβ2**			
Compound	**Tyr197 (ring)**	**Tyr204 (ring)**	**Trp156 (ring)**	**Trp156 (CO)**	**Trp57 (ring)**	**Asn109 (CO)**	**Phe119 (ring)**	**Leu121 (NH)**
(*S*)-**8**	N$^+$ (π/+)*	N$^+$ (π/+)	N$^+$ (π/+)	**NH** (HB)	-	-	Phe (π/π)	-
(*S*)-**9**	N$^+$ (π/+)*	N$^+$ (π/+)	N$^+$ (π/+)	**NH** (HB)	N$^+$ (π/+)*	**OH** (HB)	Phe (π/π)	**O**H (HB)
(*S*,*R*)-**10**	N$^+$ (π/+)*	N$^+$ (π/+)	N$^+$ (π/+)	**NH** (HB)	-	-	Phe (π/π)	-
(*S*,*S*)-**10**	N$^+$ (π/+)*	N$^+$ (π/+)	N$^+$ (π/+)	**NH** (HB)	-	-	Phe (π/π)	-
(*S*,*R*)-**11**	N$^+$ (π/+)*	N$^+$ (π/+)	N$^+$ (π/+)	**NH** (HB)	N$^+$ (π/+)*	**OH** (HB)	Phe (π/π)	**O**H (HB)
(*S*,*S*)-**11**¤	N$^+$ (π/+)*	N$^+$ (π/+)	N$^+$ (π/+)	**NH** (HB)	-	**OH** (HB)	Phe (π/π)	**O**H (HB) **Me** (clash)

¤ The methyl group of (*S*,*S*)-**11** also shows steric clashes also with the side chain of α4-Cys199.

2ifd-wc-hα4β2	**hα4**				**Water**	**hβ2**			
Compound	**Tyr197 (ring)**	**Tyr204 (ring/OH)**	**Trp156 (ring)**	**Trp156 (CO)**		**Trp57 (ring)**	**Asn109 (CO)**	**Phe119 (ring)**	**Leu121 (NH)**
(*S*)-**12**	N$^+$ (π/+)*	N$^+$ (π/+)	N$^+$ (π/+)	**NH** (HB)	**OMe** (clash)	-	**H**$_2$O (HB)	Phe (π/π)	H$_2$**O** (HB)
(*S*)-**13**¤	N$^+$ (π/+)	N$^+$ (π/+)	N$^+$ (π/+)	**NH** (HB)	**N**$_{pyr}$ (HB)*	N$^+$ (π/+)*	**H**$_2$O (HB)	Phe (π/π)	H$_2$**O** (HB)
(*S*,*R*)-**14**	N$^+$ (π/+)	N$^+$ (π/+)	N$^+$ (π/+)	**NH** (HB)*	**N**$_{pyr}$ (HB)	N$^+$ (π/+)*	**H**$_2$O (HB)	Phe (π/π)	H$_2$**O** (HB)
(*S*,*S*)-**14**▫	N$^+$ (π/+)	N$^+$ (π/+)*	N$^+$ (π/+)	**NH** (HB)*	**N**$_{pyr}$ (HB)	N$^+$ (π/+)	**H**$_2$O (HB)	Phe (π/π)	H$_2$**O** (HB)

¤ The pyridine ring of (*S*)-**12** shows unfavorable steric contacts with the side chain of *α*4-Thr157.

▫ The methyl group of (*S*,*S*)-**14** clashes with the side chain of α4-Cys199.

29ifd-wc-hα3β4	**hα3**				**Water**	**hβ4**			
Compound	**Tyr190 (ring)**	**Tyr197 (ring)**	**Trp149 (ring)**	**Trp149 (CO)**		**Trp59 (ring)**	**Asn111 (CO)**	**Leu121 (s.chain)**	**Leu123 (NH)**
(*S*,*R*)-**14**	N$^+$ (π/+)*	N$^+$ (π/+)	N$^+$ (π/+)	**NH** (HB)*	-	N$^+$ (π/+)	**H**$_2$O (HB)	-	H$_2$**O** (HB)

Table S4. Overview of the relevant interactions of the compounds (*S*)-**15** and (*S*)-**16** reported in Figure 8. The substructure of the residue involved in the interaction is reported in brackets. The atom or group of the ligand interacting with each residue is highlighted in bold. The type of interaction is reported in brackets. Interactions marked with * are not explicitly visualized, but at a suitable distance for interaction, very close to the default visualization thresholds (< 7 Å for π/+ and < 3.2 Å for H-bonds).

16ifd-wf-hα4β2		**hα4**				**hβ2**			
Compound	**Tyr197 (ring)**	**Tyr204 (ring)**	**Trp156 (ring)**	**Trp156 (CO)**	**Glu102 (COO^-)**	**Asn109 (CO)**	**Phe119 (ring)**	**Leu121 (NH)**	**Lys79 (NH_3^+)**
(*S*)-**15**	N^+ (π/+)	N^+ (π/+)	N^+ (π/+)	N**H** (HB)	**OH**$_{\text{Hexyn-1-ol}}$ (HB)	O**H**$_{\text{Phe}}$ (HB)	Phe (π/π)*	**O**H$_{\text{Phe}}$ (HB)	**O**H$_{\text{Hexyn-1-ol}}$ (HB)*
(*S*)-**16**	N^+ (π/+)	N^+ (π/+)	N^+ (π/+)	N**H** (HB)	O**H**$_{p\text{-OH-Ph}}$ (HB)	O**H**$_{\text{Phe}}$ (HB)	Phe (π/π)* *p*-OH-**Ph**(π/π)*	**O**H$_{\text{Phe}}$ (HB)	O**H**$_{p\text{-OH-Ph}}$ (HB)*

16ifd-wf-hα3β4	**hα3**					**hβ4**			
Compound	**Tyr190 (ring)**	**Tyr197 (ring)**	**Trp149 (ring)**	**Trp149 (CO)**	**Glu194 (COO^-)**	**Asn111 (CO)**	**Leu119 (s.chain)**	**Leu123 (NH)**	**Arg115 (NH_2^+)**
(*S*)-**15**¤	N^+ (π/+)	N^+ (π/+)	N^+ (π/+)	N**H** (HB)*	-	O**H**$_{\text{Phe}}$ (HB)	-	**O**H$_{\text{Phe}}$ (HB)	CH_2 (clash)
(*S*)-**16**	N^+ (π/+)*	N^+ (π/+)	N^+ (π/+)	N**H** (HB)*	O**H**$_{p\text{-OH-Ph}}$ (HB)	O**H**$_{\text{Phe}}$ (HB)	-	**O**H$_{\text{Phe}}$ (HB)	O**H**$_{p\text{-OH-Ph}}$ (HB)*

¤ the hexyn-2-ol group of (*S*)-**15** also clashes with the side chain of β4-Lys61.

`

Table S5. Overview of the relevant interactions of the compounds (*S*,*R*)-**2**, (*S*,*R*)-**26,** (*S*,*R*)-**29,** (*S*,*R*)-**31** and (*S*,*S*)-**37-40** reported in Figure 9 and Figure 11, respectively. The substructure of the residue involved in the interaction is reported in brackets. The atom of the ligand interacting with each residue is highlighted in bold. The type of interaction is reported in brackets. Interactions marked with * are not explicitly visualized, but at a suitable distance and angle for interaction, very close to the default visualization thresholds (< 7 Å for π/+ and < 3.2 Å for H-bonds).

2ifd-wf-hα4β2	**hα4**				**hβ2**			
Compound	**Tyr197 (ring)**	**Tyr204 (ring)**	**Trp156 (ring)**	**Trp156 (CO)**	**Trp57 (ring)**	**Asn109 (CO)**	**Phe119 (ring)**	**Leu121 (NH)**
(*S*,*R*)-**2**	N$^+$ (π/+)	N$^+$ (π/+)*	N$^+$ (π/+)	**NH** (HB)	N$^+$ (π/+)*	**OH** (HB)	Phe (π/π)	**O**H (HB)
(*S*,*R*)-**26**¤	N$^+$ (π/+)	N$^+$ (π/+)*	N$^+$ (π/+)	**NH** (HB)	N$^+$ (π/+)	-	Phe (π/π)	O$\mathbf{CH_3}$ (clash)
(*S*,*R*)-**29**	N$^+$ (π/+)	N$^+$ (π/+)	N$^+$ (π/+)	**NH** (HB)	N$^+$ (π/+)*	N$\mathbf{H_2}$ (HB)	Phe (π/π)	**N**H$_2$ (HB)
(*S*,*R*)-**31**¤	N$^+$ (π/+)	N$^+$ (π/+)*	N$^+$ (π/+)	**NH** (HB)*	N$^+$ (π/+)*	N$\mathbf{Me_2}$ (clash)	Phe (π/π)	**N**Me$_2$ (HB)*
(*S*,*S*)-**37**▫	N$^+$ (π/+)*	N$^+$ (π/+)	N$^+$ (π/+)	**NH** (HB)	N$^+$ (π/+)*	N$\mathbf{H_2}$ (HB)	Phe (π/π)*	**N**H$_2$ (HB)*
(*S*,*S*)-**38**◊	N$^+$ (π/+)*	N$^+$ (π/+)	N$^+$ (π/+)	**NH** (HB)	N$^+$ (π/+)*	-	Phe (π/π)	N$\mathbf{O_2}$(HB)
(*S*,*S*)-**39**▫	N$^+$ (π/+)*	N$^+$ (π/+)	N$^+$ (π/+)	**NH** (HB)*	N$^+$ (π/+)*	-	Phe (π/π)	**O**H (HB)*
(*S*,*S*)-**40**	N$^+$ (π/+)	N$^+$ (π/+)	N$^+$ (π/+)	**NH** (HB)*	N$^+$ (π/+)*	-	Phe (π/π)*	-
(*S*,*R*)-**43**	N$^+$ (π/+)	N$^+$ (π/+)	N$^+$ (π/+)	-	N$^+$ (π/+)*	**OH** (HB)	Phe (π/π)	-

¤ The methyl group of (*S*,*R*)-**26** and of (*S*,*R*)-**31** also clash with the side chain of β2-Ser108.
▫ The amino group of (*S*,*S*)-**37** and the hjydroxyl group of (*S*,*S*)-**39** also interact (H-bond) with the backbone carbonyl of β2-Leu121.
◊ The nitro group of (*S*,*S*)-**38** also interacts (H-bond) with the side chain of β2-Ser108.

29ifd-wf-hα3β4	**hα3**				**hβ4**			
Compound	**Tyr190 (ring)**	**Tyr197 (ring)**	**Trp149 (ring)**	**Trp149 (CO)**	**Trp59 (ring)**	**Asn111 (CO)**	**Leu119 (s.chain)**	**Leu123 (NH)**
(*S*,*R*)-**2**	N$^+$ (π/+)*	N$^+$ (π/+)	N$^+$ (π/+)	**NH** (HB)	N$^+$ (π/+)	**OH** (HB)	-	**O**H (HB)
(*S*,*R*)-**26**	N$^+$ (π/+)*	N$^+$ (π/+)	N$^+$ (π/+)	**NH** (HB)	N$^+$ (π/+)	$\mathbf{OCH_3}$ (clash)	-	**O**CH$_3$ (HB)
(*S*,*R*)-**29**	N$^+$ (π/+)*	N$^+$ (π/+)	N$^+$ (π/+)	**NH** (HB)	N$^+$ (π/+)	N$\mathbf{H_2}$ (HB)	-	**N**H$_2$ (HB)*
(*S*,*R*)-**31**	N$^+$ (π/+)*	N$^+$ (π/+)	N$^+$ (π/+)	**NH** (HB)	N$^+$ (π/+)	-	-	-
(*S*,*R*)-**43**	N$^+$ (π/+)*	N$^+$ (π/+)	N$^+$ (π/+)	**NH** (HB)	N$^+$ (π/+)	-	-	-

`

Table S6. Overview of the relevant interactions of the compounds (*S,R*)-**33-36** reported in Figure 10. The substructure of the residue involved in the interaction is reported in brackets. The atom of the ligand interacting with each residue is highlighted in bold. The type of interaction is reported in brackets. Interactions marked with * are not explicitly visualized, but at a suitable distance and angle for interaction, very close to the default visualization thresholds (< 7 Å for π/+ and < 3.2 Å for H-bonds).

2ifd-wc-hα4β2	**hα4**					**hβ2**			
Compound	**Tyr197 (ring)**	**Tyr204 (ring/OH)**	**Trp156 (ring)**	**Trp156 (CO)**	**Water**	**Trp57 (ring)**	**Asn109 (CO)**	**Phe119 (ring)**	**Leu121 (NH)**
(*S*,*R*)-**33**	N^+ (π/+)	N^+ (π/+)	N^+ (π/+)	N**H** (HB)*	**N**$_{pyr}$ (HB)	N^+ (π/+)*	**H**$_2$O (HB)	Pyr (π/π)	H$_2$**O** (HB)
(*S*,*R*)-**34**	N^+ (π/+)	N^+ (π/+)	N^+ (π/+)	-	Pyr (clash)	N^+ (π/+)*	**H**$_2$O (HB)	Pyr (π/π)	H$_2$**O** (HB)
(*S*,*R*)-**35**	N^+ (π/+)	N^+ (π/+)*	N^+ (π/+)	N**H** (HB)*	**N**$_{pyr}$ (HB)<br>Pyr (clash)	N^+ (π/+)*	**H**$_2$O (HB)	Pyr (π/π)*	H$_2$**O** (HB)
(*S*,*R*)-**36**	N^+ (π/+)*	N^+ (π/+)	N^+ (π/+)	-	**O**$_{diox}$	N^+ (π/+)*	**H**$_2$O (HB)	Pyr (π/π)	H$_2$**O** (HB)

29ifd-wc-hα3β4	**hα3**					**hβ4**			
Compound	**Tyr190 (ring)**	**Tyr197 (ring)**	**Trp149 (ring)**	**Trp149 (CO)**	**Water**	**Trp59 (ring)**	**Asn111 (CO)**	**Leu121 (s.chain)**	**Leu123 (NH)**
(*S*,*R*)-**33**	N^+ (π/+)*	N^+ (π/+)	N^+ (π/+)	N**H** (HB)	**O**$_{diox}$	N^+ (π/+)	**H**$_2$O (HB)	-	H$_2$**O** (HB)

Table S7. RMSD values from the self-docking study, calculated between the native ligand and the re-docked ligand for each prepared binding site.

**Binding site**	**Native Ligand**	**Reference figures**	**RMSD**
hα4β2 (6CNJ)– water	NIC	Fig 4A, 6A	0,6287
hα3β4 (6PV7) – water	NIC	Fig 4A, 6B	1,4374
hα3β4 (6PV8) – AB interface	AT-1001	Fig 4B	0,5057
hα3β4 (6PV8) – DE interface	AT-1001	Fig 4C	0,7287
hα4β2 (6CNJ) – no water	NIC	Fig 5, 6A	0,6529
hα3β4 (6PV7) – no water	NIC	Fig 5, 6B	0,7641
hα4β2 adapted on (S,R)-2 – water	NIC	Fig 7C, 10, 11A	1,0079
hα4β2 adapted on (S,R)-2 – no water	NIC	Fig 7A, 7B, 9A, 11A-C	1,1029
hα3β4 adapted on (S,R)-29 – water	NIC	Fig 7D, 10B	1,1595
hα3β4 adapted on (S,R)-29 – no water	NIC	Fig 9B, 11D	1,1466
hα4β2 adapted on (S)-16	NIC	Fig 8A	1,9651
hα3β4 adapted on (S)-16	NIC	Fig 8B	1,2641
